# Transcutaneous Auricular Vagus Nerve Stimulation Modulating the Brain Topological Architecture of Functional Network in Major Depressive Disorder: An fMRI Study

**DOI:** 10.3390/brainsci14090945

**Published:** 2024-09-21

**Authors:** Zhi-Peng Guo, Dan Liao, Lei Chen, Cong Wang, Miao Qu, Xue-Yu Lv, Ji-Liang Fang, Chun-Hong Liu

**Affiliations:** 1 Beijing Hospital of Traditional Chinese Medicine, Capital Medical University, Beijing 100010, China; zhipengguo0620@163.com (Z.-P.G.); chenlei9449@163.com (L.C.); 2 Department of Radiology, Guizhou Provincial People’s Hospital, Guiyang 550002, China; liaodann@163.com; 3 Kerfun Medical (Suzhou) Co., Ltd., Suzhou 215000, China; 13991858677@163.com; 4 Department of Neurology, Xuanwu Hospital, Capital Medical University, Beijing 100053, China; qumiao@xwhosp.org; 5 Guang’anmen Hospital, China Academy of Chinese Medical Sciences, Beijing 100053, China; yuyu1208@163.com (X.-Y.L.); fangmgh@163.com (J.-L.F.)

**Keywords:** major depression disorder, transcutaneous auricular vagus nerve stimulation, graph theory, network-based statistics, default mode network

## Abstract

Background: Transcutaneous auricular vagus nerve stimulation (taVNS) is effective in regulating mood and high-level cognition in patients with major depressive disorder (MDD). This study aimed to investigate the efficacy of taVNS treatment in patients with MDD and an altered brain topological organization of functional networks. Methods: Nineteen patients with MDD were enrolled in this study. Patients with MDD underwent 4 weeks of taVNS treatments; resting-state functional magnetic resonance imaging (rs-fMRI) data of the patients were collected before and after taVNS treatment. The graph theory method and network-based statistics (NBS) analysis were used to detect abnormal topological organizations of functional networks in patients with MDD before and after taVNS treatment. A correlation analysis was performed to characterize the relationship between altered network properties and neuropsychological scores. Results: After 4 weeks of taVNS treatment, patients with MDD had increased global efficiency and decreased characteristic path length (Lp). Additionally, patients with MDD exhibited increased nodal efficiency (NE) and degree centrality (DC) in the left angular gyrus. NBS results showed that patients with MDD exhibited reduced connectivity between default mode network (DMN)–frontoparietal network (FPN), DMN–cingulo-opercular network (CON), and FPN–CON. Furthermore, changes in Lp and DC were correlated with changes in Hamilton depression scores. Conclusions: These findings demonstrated that taVNS may be an effective method for reducing the severity of depressive symptoms in patients with MDD, mainly through modulating the brain’s topological organization. Our study may offer insights into the underlying neural mechanism of taVNS treatment in patients with MDD.

## 1. Introduction

Major depressive disorder (MDD) is a common psychiatric disorder that affects over 185 million people globally and is expected to be the leading cause of the global burden of mental illness by 2030 [1]. However, current treatments for MDD are unsatisfactory. The most common treatment of MDD is pharmacotherapy using antidepressants, and only approximately one-third of patients with MDD are responsive to drug therapy. Moreover, most patients with MDD are at risk of drug dependence after long-term use of antidepressants [2,3]. Other non-drug therapies, such as transcranial magnetic stimulation (TMS) [4], transcranial direct current stimulation (tDCS) [5], deep brain stimulation (DBS) [6], and electroconvulsive therapy (ECT) [7], have been increasingly used for the treatment of MDD. However, these treatment options are difficult to implement widely in clinical practice, mainly due to their poorly understood side effects, complex procedures, and high costs [8].

The vagus nerve is the longest cranial nerve in the human body and has an important role in the regulation of several body systems. It plays a key role in maintaining the stability of the internal environment, including physiological and biochemical processes [9]. The auricular branch of the vagus nerve is its only cutaneous branch [10]. Researchers have suggested that electric acupuncture stimulating the external ear canal, which is innervated by the cutaneous branch of the vagus nerve, produces a “vagus nerve effect”, which can improve the clinical symptoms of MDD [11,12]. Transcutaneous auricular VNS (taVNS), as proposed by Ventureyra in 2000 [13], is effective in treating disorders such as epilepsy [14], migraines [3], insomnia [15], and MDD [16]. The basis of taVNS as a symptomatic treatment in various neuropsychiatric disorders may lie in its capacity to modulate diffuse neuromodulatory systems, including the noradrenergic, cholinergic, and serotonergic systems [17]. Furthermore, taVNS does not only reduce the shortcomings (e.g., invasive operation and high cost) of VNS but is also simple and easy to perform [10]. Similar to taVNS, TMS is noninvasive and has been used to improve symptoms of depression [18] by employing a pulsed magnetic field to induce an electrical current and modulate the activities of some brain regions. Previous studies have also shown that taVNS can significantly reduce the severity of depressive symptoms in patients with MDD [11,12,19]. However, although taVNS may be a promising and effective method for the management of MDD, its potential mechanism remains unknown.

Resting-state functional magnetic resonance imaging (rs-fMRI) is a classic tool for assessing the neural activity of the brain using blood oxygen level-dependent (BOLD) signals. These approaches have been widely utilized to investigate the underlying neural mechanism of insomnia [20], MDD [21], and the treatment efficiency of taVNS [3,8]. Sun et al. found that after 30 min of taVNS treatments, patients with MDD displayed improvements in symptoms of depression, mainly reflected by the decreased amplitude of low-frequency fluctuations (ALFFs) in the right precuneus and decreased functional connectivity (FC) between the default mode network (DMN) and the frontoparietal network (FPN) [8]. Yi et al. found that after 4 weeks of taVNS treatments, patients with MDD who had a history of suicidal attempts experienced improvement in symptoms of depression, reflected by a decrease in regional homogeneity (ReHo) in the left supplementary motor area and the right median cingulate cortex, and a decrease in ReHo values that were correlated with changes in Hamilton depression (HAMD) scores [3]. Therefore, taVNS could reduce the severity of depressive symptoms by regulating spontaneous brain activity.

As a complex hierarchical network, the human brain is capable of efficient information segmentation and integration [22]. In recent years, the advent of network science in the field of neuroscience has offered a novel approach for the topological characterization of the functional bases that underpin brain activities [23]. Numerous neuroimaging studies have emphasized the combination of rs-fMRI and graph theory analysis [24]. The graph theory method mathematically defines the entire brain as a graph, consisting of nodes (brain regions) and edges (the connectivity between any pair of brain regions) [25], and can be used to detect the transmission of information across the entire brain. Network-based statistics (NBS) analysis is another graph theory tool that provides a complementary approach for recognizing the connected subnetworks and the underlying feature of these networks [26].

Previous studies have indicated that the topological properties of the brain were altered in patients with MDD. Zhang et al. found that patients with MDD showed reduced global and local efficiency compared with controls [27]. Jacob et al. found that patients with MDD showed decreased levels of centrality (DC) in the right precuneus [28]. Furthermore, Dai et al. found that after 8 weeks of treatment using antidepressants, patients with MDD exhibited decreased topological properties (characteristic path length and the clustering coefficient) and decreased characteristic path length that correlated with the subscale of HAMD scores [29]. Wu et al. revealed that after treatment using ECT, patients with MDD exhibited increased DC in the bilateral angular cortex (AG), precuneus, and right superior frontal gyrus [30]. However, to the best of our knowledge, the topological reorganization in patients with MDD after taVNS treatment has not been investigated, and this may limit the understanding and interpretation of the neural mechanisms in patients with MDD after taVNS treatment.

In this study, we hypothesized that taVNS treatment is effective in reducing the severity of depressive symptoms in patients with MDD through the modulation of the topological organization of the brain. To test this hypothesis, the graph theory method and NBS analysis were performed in patients with MDD before and after treatment using taVNS. We also explored the relationships between changed network topological properties and a changed neuropsychological scale. Our study may provide new insights into the underlying brain mechanism for taVNS treatment in patients with MDD.

## 2. Materials and Methods

### 2.1. Participants

Nineteen patients with MDD were recruited from the Psychiatry Department of Guang’anmen Hospital. Due to safety and ethical concerns, we only recruited patients with mild or moderate MDD. No participant had suicidal ideation before or after treatment. Patients with MDD were diagnosed according to the American Diagnostic and Statistical Manual of Mental Disorders, Fifth Edition (DSM-5) by two qualified psychiatrists using a clinically structured interview. This study was approved by the Ethics Committee of the Guang’anmen Hospital, Beijing Hospital of Traditional Chinese Medicine. Written informed consent was obtained from each participant before being enrolled in the study.

The G*Power (version 3.1.9.7) was used to calculate the sample size. A previous study reported an effect size of 0.57 [11]. Based on a modest power (1—beta = 0.8) and the standard alpha level (0.05, two-tailed), 15 patients with MDD were needed. Considering the loss of visits and refusals (20%), at least 19 patients with MDD were needed.

The inclusion criteria for patients with MDD were as follows: (1) patients who were willing to receive taVNS treatment; (2) HAMD scores > 17; (3) age between 18 and 60 years; (4) right-handedness; (5) patients willing to discontinue the use of antidepressants or other psychiatric drugs two weeks before beginning the treatment; and (6) patients who had exhibited depressive symptoms for at least two weeks.

The exclusion criteria for patients with MDD were as follows: (1) patients with other psychiatric disorders (such as bipolar disorder, etc.); (2) pregnancy; (3) contraindications to MRI; (4) patients with a history of drug and/or alcohol abuse; and (5) history of severe medical diseases.

### 2.2. TaVNS Treatment

**Figure 1:**
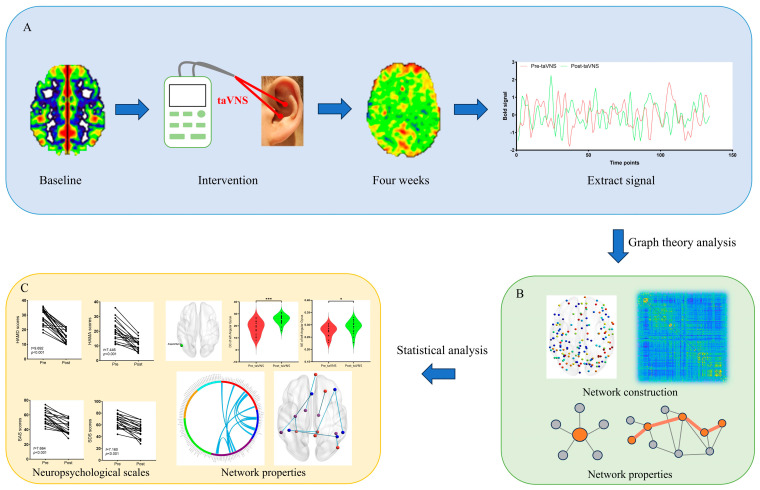
Study workflow. (**A**) taVNS intervention and bold signal extraction. (**B**) Graph theory analysis. (**C**) Statistical analysis. Abbreviations: taVNS, transcutaneous auricular vagus nerve stimulation. * *p* < 0.05, *** *p* < 0.001.

In this study, all patients with MDD received 4 weeks of taVNS treatment, and a taVNS device (Hwato, SDZ-IIB, Suzhou, China) was used to stimulate the auricular concha area where there is an abundant branch distribution of the vagus nerve (Figure 1).

The stimulation parameters of taVNS were as follows: (1) the intensity is 4–6 milliamperes (adjusted according to the tolerance of patients); (2) the frequency is 20 Hz, with a wave width of <1 millisecond; and (3) each treatment lasted for 30 min and was performed twice a day (morning and evening) for a minimum of five days per week.

### 2.3. Neuropsychological Scales

The participants were evaluated using the Hamilton Anxiety Rating Scale (HAMA), the 24-item Hamilton Depression Rating Scale, the Self-Rating Anxiety Scale (SAS), and the Self-Rating Depression Scale (SDS) before and after 4 weeks of taVNS treatments by qualified psychiatrists. The severity of depressive symptoms was assessed by the HAMD score [31]. The severity of anxiety symptoms was detected using the HAMA score [32]. The SDS and SAS scores were used to measure the depressive and anxiety symptoms [33].

### 2.4. MRI Data Acquisition

The functional MR data were acquired from a GE Signa 1.5 Tesla scanner (GE Healthcare, Buckinghamshire, UK) at the Guang’anmen Hospital. All participants underwent rs-fMRI scans before and after the 4-week treatment of taVNS. During the MRI scans, the participants were instructed to keep their eyes closed without falling asleep and to have relaxed thoughts while refraining from engaging in any specific thought.

The rs-fMRI data were acquired with the single-shot gradient-echo EPI sequence as follows: echo time (TE) = 30 ms, repetition time (TR) =2500 ms, field of view (FOV) = 240 × 240 mm^2^, fip angle (FA) = 90°, gap = 0.5 mm, matrix = 64 × 64, slice thickness = 3.0 mm, and number of slices = 41. The parameters of T1-weighted images were as follows: TR = 8.1 ms, TE = 3.7 ms, FA = 8°, number of slices = 170, FOV = 256 × 256 mm^2^, slice thickness = 1 mm, and matrix = 256 × 256. The rs-fMRI sequence scan took 8 min and 6 s.

### 2.5. Data Preprocessing

The rs-fMRI data were preprocessed by using the graph theoretical network analysis toolbox (GRETNA, http://www.nitrc.org/projects/gretna/ (accessed on 25 September 2021)) and Statistical Parametric Mapping, version 12 (SPM12, http://www.fil.ion.ucl.ac.uk/spm/ (accessed on 13 August 2021)) [34]. The steps of data preprocessing were as follows: (1) the first 10 volumes were removed because of possible equilibration of the magnetic field and movement; (2) slice timing and realignment of head motion were performed in the remaining 133 time points; participants with a head motion (<2 mm or <2°) were excluded; (3) diffeomorphic anatomical registration through the exponentiated lie algebra (DARTEL) were used to normalize the generated images into the Montreal Neurological Institute (MNI) space and then resampled to a 3 × 3 × 3 mm isotropic voxel size; (4) to reduce the influence of MRI equipment, linear detrending was performed; (5) to reduce the physiological high-frequency respiratory and cardiac noise, low-frequency band-pass filtering (0.01–0.10 Hz) was performed; (6) a Gaussian kernel with a full width at half maximum of 6 mm was used for spatial smoothing; and (7) the framewise displacement values were also calculated to minimize the possible effects of micromovements of the head.

### 2.6. Functional Network Construction

As the basic elements of a network, the definition of the nodes and edges was key to the construction of the network. In the current study, the brain network nodes were defined by the Dosenbach atlas, which includes 160 cortical or subcortical regions [35]. Afterwards, Pearson correlation was employed to determine the edges of the network, which was calculated based on the mean time series of each node pair. Subsequently, a 160 × 160 functional connectivity (FC) matrix was obtained for each participant and then transformed into a binary matrix through a set of thresholds.

Consistent with the methods used in previous studies, our study utilized a sparsity range (0.05–0.4) with a step of 0.01 to construct the brain network [36]. The ratio of edges in a network compared with the maximum possible number of edges was the sparsity value. The minimum sparsity was the lower bound when all the nodes were connected in a network. Finally, we performed Fisher’s z-transformation on the individual matrix.

### 2.7. Network Metrics Analysis

Global and nodal network properties of the brain network were calculated at a serial of the sparsity using the GRETNA toolbox. Global network properties included the following: clustering coefficient (Cp), normalized Cp (γ), characteristic path length (Lp), normalized Lp (λ), small-worldness (σ), global efficiency (Eglob), and local efficiency (Eloc). The nodal network properties included the betweenness centrality (BC), degree centrality (DC), and nodal efficiency (NE) [36].

In order to offer a summarized scalar independent of single-threshold selection, the area under the curve (AUC) of topological properties were calculated. Detailed information is presented in Table S1.

### 2.8. Statistical Analysis

SPSS version 25.0 software (IBM Corp., Armonk, NY, USA) was used to compare demographic and clinical data, and the normality of the distribution was detected using the Kolmogorov–Smirnov test. A paired *t*-test was used to detect the alternation of the neuropsychological scale in patients with MDD before and after taVNS treatment, and *p* < 0.05 was used as the threshold of statistical significance.

Global and nodal properties were analyzed using the GRETNA toolbox. Paired *t*-tests were used to detect differences in patients with MDD before and after taVNS treatment, with covariates (age, sex, and mean FD values). The false discovery rate (FDR corrected, *p* < 0.05) was used for multiple comparisons.

#### 2.8.1. NBS Analysis

NBS analysis was used to further investigate the edge-based FC of the network in patients with MDD before and after taVNS treatment. This analysis was performed using the NBS method (version 1.2, https://www.nitrc.org/projects/nbs (accessed on 13 August 2021)) [37], which offers a higher statistical power [38]. The steps involved were as follows: (1) the t-statistic was used to determine a range of suprathreshold links for each network edge; (2) connected components were detected using a breadth-first search, where any two regions were linked by suprathreshold connections; (3) random permutation testing (5000 times) was conducted with the *p*-value controlled for a family-wise error, which was attributed to the number of links in each connected component; and (4) a corrected *p*-value was obtained by computing the proportion of permutations, as the null distribution for the size of the largest connected component was generated during the permutation process.

#### 2.8.2. Correlation Analysis

We also conducted correlation analyses between altered topological properties and the change in neuropsychological scale (HAMD, HAMA, SAD, and SDS) scores. The threshold of statistical significance was *p* < 0.05.

## 3. Results

### 3.1. Demographic and Neuropsychological Scores

The demographic and neuropsychological scores of participants are depicted in Table 1. After 4 weeks of taVNS treatment, there were significant differences between pre- and post-treatment HAMD, HAMA, SAS, and SDS scores of patients with MDD (all *p* < 0.001). These changes in neuropsychological scales are shown in Table 2 and Figure S1.

### 3.2. Global Topological Properties

**Figure 2:**
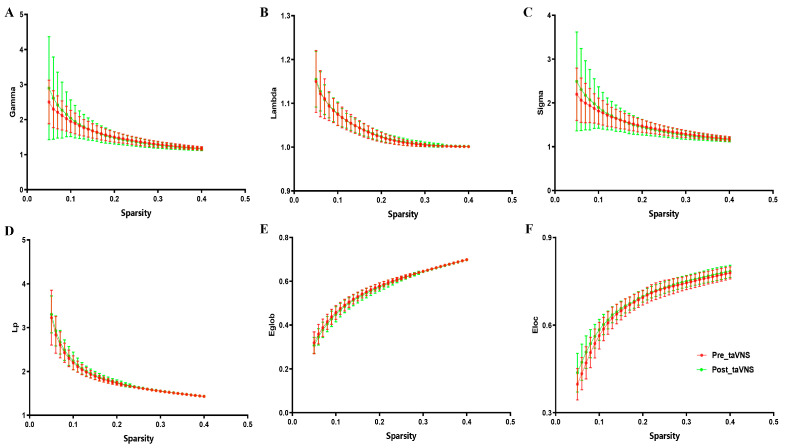
Differences in global network topological properties of patients with MDD across the sparsity range (0.05–0.4). (**A**) Gamma in patients with MDD before and after taVNS treatment; (**B**) lambda in patients with MDD; (**C**) sigma in patients with MDD; (**D**) Lp in patients with MDD; (**E**) Eglob in patients with MDD; (**F**) Eloc in patients with MDD. Abbreviations: Lp, characteristic path length; MDD, major depressive disorder; Eglob, global efficiency; taVNS, transcutaneous auricular vagus nerve stimulation; Eloc, local efficiency.

**Figure 3:**
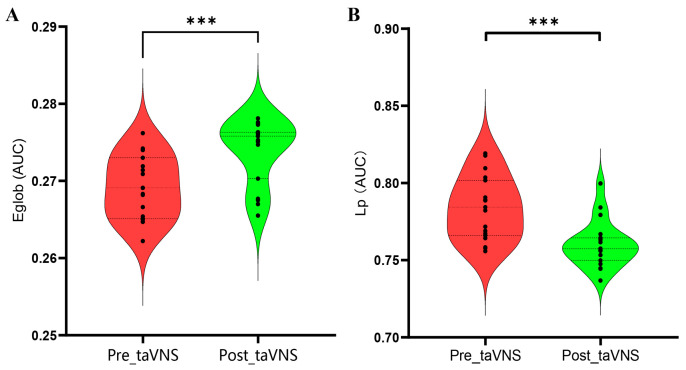
Differences in global properties of patients with MDD before and after taVNS treatment based on AUC values. (**A**) Eglob in patients with MDD; (**B**) Lp in patients with MDD. Abbreviations: Eglob, global efficiency; taVNS, transcutaneous auricular vagus nerve stimulation; Lp, characteristic path length; MDD, major depressive disorder; AUC, area under the curve. *** *p* < 0.001.

Patients with MDD (before and after the taVNS treatment) all demonstrated small-worldness (sigma > 1), with gamma > 1 and lambda approximately equal to one across the sparsity range of 0.05–0.40 (step = 0.01) (Figure 2). After 4 weeks of taVNS treatment, patients with MDD demonstrated increased Eglob and decreased Lp compared to baseline values (*p* < 0.001, FDR corrected). No significant difference was observed in other global properties. Detailed information is presented in Table 3 and Figure 3.

### 3.3. Nodal Topological Properties

**Figure 4:**
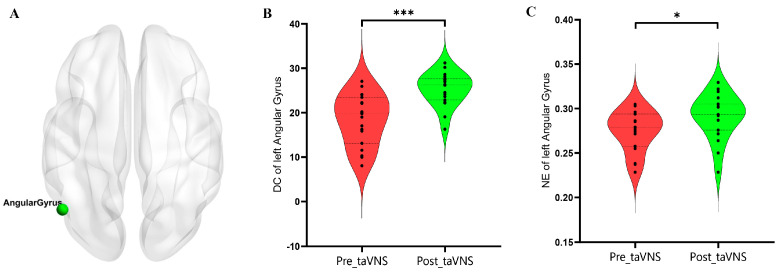
Differences in nodal properties of patients with MDD before and after taVNS treatment based on the AUC values. (**A**) The left AG; (**B**) the DC of the left AG in patients with MDD; (**C**) the NE of the left AG in patients with MDD. Abbreviations: AG, angular gyrus; AUC, area under the curve; DC, degree centrality; taVNS, transcutaneous auricular vagus nerve stimulation; NE, nodal efficiency; MDD, major depressive disorder. * *p* < 0.05, *** *p* < 0.001.

After 4 weeks of taVNS treatment, the patients with MDD showed significantly increased DC values in the left angular gyrus (AG) (*p* < 0.001, FDR corrected). In addition, the patients with MDD showed significantly increased NE values of the left AG (*p* = 0.048, FDR corrected). The changes in the participants’ global network topological properties are presented in Table 3 and Figure 4.

### 3.4. NBS Analysis

**Figure 5:**
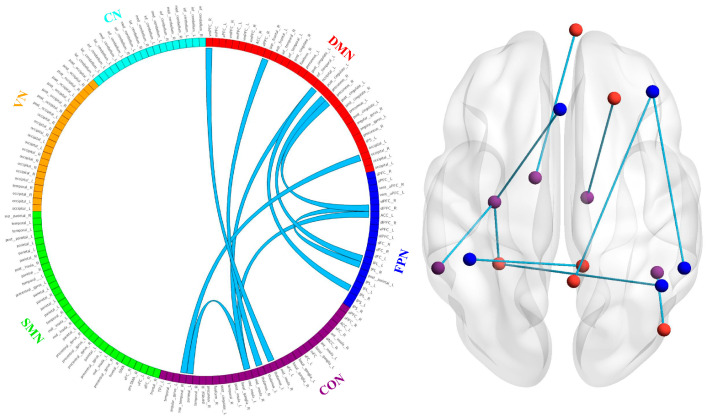
Differences in network connectivity of patients with MDD before and after taVNS treatment. The blue lines indicate the decreased functional connections between the DMN and FPN, DMN and CON, and FPN and CON. Abbreviations: CON, cingulo-opercular network; CN, cerebellar network; DMN, default mode network; MDD, major depressive disorder; FPN, frontoparietal network; SMN, sensorimotor network; taVNS, transcutaneous auricular vagus nerve stimulation; VN, visual network.

Compared with baseline values, after 4 weeks of taVNS treatment, patients with MDD showed decreased FC in several regions, which contained 16 nodes and 10 edges (*p* < 0.05, FDR corrected), mainly relating to the default mode network (DMN), frontoparietal network (FPN), and cingulo-opercular network (CON). These edges connected the left thalamus and the right ventromedial prefrontal cortex (vmPFC), the right thalamus to the right superior frontal cortex, the right inferior parietal lobe and left middle insula to the left occipital cortex, the left inferior parietal lobe to the right precuneus, the right dorsolateral prefrontal cortex (dlPFC) to the right precuneus, the right superior temporal cortex to the right occipital cortex, the right inferior parietal lobe to the right dlPFC, the left middle insula to the left anterior cingulate cortex, and the left middle insula to the left parietal lobe. Specifically, after taVNS treatment, patients with MDD exhibited reduced connectivity between the DMN–FPN, DMN–CON, and FPN–CON (all *p* < 0.05, FDR corrected). The visualization of these alterations in FC is shown in Figure 5.

### 3.5. Correlation Analysis

**Figure 6:**
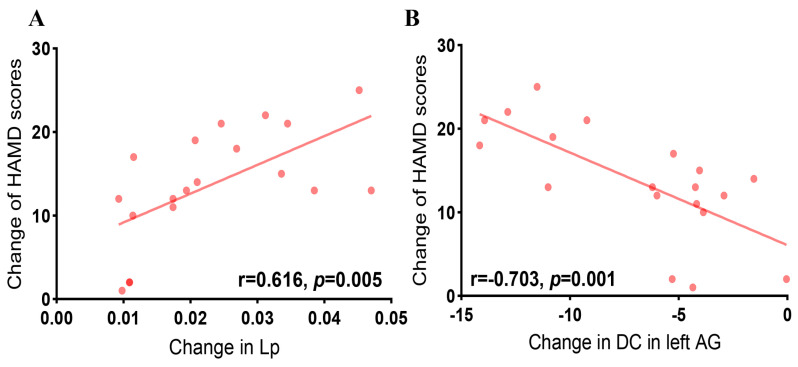
Relationships between changes in global and nodal network properties and changes in HAMD scores of patients with MDD after taVNS treatment. (**A**) Change in Lp was positively associated with alternation in HAMD scores (r = 0.616, *p* = 0.005). (**B**) Changes in DC in the left AG were negatively associated with changes in HAMD scores (r = −0.703, *p* = 0.001). Abbreviations: AG, angular gyrus; DC, degree centrality; MDD, major depressive disorder; HAMD, 24-Hamilton Depression Rating Scale; Lp, characteristic path length; taVNS, transcutaneous auricular vagus nerve stimulation.

The correlation analysis indicated that the change in Lp was associated with changes in HAMD scores in patients with MDD (r = 0.616, *p* = 0.005). Furthermore, changes in DC in the left AG were negatively associated with changes in HAMD scores (r = −0.703, *p* = 0.001) (Figure 6).

## 4. Discussion

By combining the graph theory method and NBS analysis, our study investigated topological metrics and FC changes in patients with MDD before and after taVNS treatment. After 4 weeks of taVNS treatment, patients with MDD had increased Eglob and decreased Lp. Additionally, patients with MDD exhibited increased DC and NE in the left AG and reduced FC between the DMN–FPN, DMN–CON, and FPN–CON. The alternations in Lp and DC were correlated with changes in HAMD scores. Our study may offer insights into the underlying neural mechanisms of taVNS treatment in patients with MDD.

### 4.1. TaVNS Treatment Efficiency

In this study, after 4 weeks of treatment using taVNS, patients with MDD exhibited significantly decreased HAMD scores, which indicated improvements in depressive symptoms. Our study’s results are consistent with those of previous studies that concluded that depressive symptoms were relieved in patients with MDD after treatment with taVNS [11,39]. Liu et al. found that after 4 weeks of taVNS treatments, patients with MDD showed significantly decreased HAMD scores [39]. Rong et al. also found that after 4 weeks of taVNS treatment, patients with MDD demonstrated significantly reduced HAMD scores [11]. To summarize, our findings, together with those of the previous literature, indicate that the taVNS method may be an effective tool to relieve the depressive symptoms in patients with MDD.

### 4.2. Global Topological Properties’ Alteration after taVNS Treatment

A brain with small-worldness is associated with a high Cp and a low Lp, supporting the balance of two fundamental brain organization principles, namely segregation and integration [40]. These properties facilitate the efficient transfer of information throughout the brain’s network with minimal wiring and energy [41]. In the current study, patients with MDD (both before and after taVNS treatment) exhibited small-worldness, indicating that the small-world model may not be easily changed after taVNS treatment in order to ensure an accurate transmission of information throughout the entire brain [42].

The Eglob and Lp represent the capacity of global information transmission in a brain network, and a high Eglob and low Lp indicate stronger information integration and faster information communication [22,29]. Daws et al. found that after psilocybin therapy, patients with MDD experienced enhanced global integration [43]. Dai et al. revealed that after 9 weeks of treatment using antidepressants, the Eglob was increased while the Lp was decreased in patients with MDD [29]. In the current study, the findings showed that patients with MDD had increased Eglob and decreased Lp after 4 weeks of taVNS treatment; these findings are consistent with those of previous studies [42,44,45].

Furthermore, changes in Lp were associated with changes in HAMD scores in patients with MDD, suggesting that the change in Lp was related to improvement in the severity of depressive symptoms in patients with MDD. Zhang et al. observed that after 8 weeks of treatment using antidepressants, changes in Lp were associated with a reduction in HAMD scores in patients with MDD [46]. This suggests that taVNS could improve the depressive symptoms in patients with MDD by normalizing the disrupted network global topological organization.

### 4.3. Nodal Topological Properties’ Alteration after taVNS Treatment

Within the mathematical framework of graph theory, DC is often used to quantify the functional connectivity of a node [47]. Higher DC values represent a stronger influence on other nodes and a greater capacity to communicate information in the network, making it potentially a good target for intervention at the nodal level [48]. In addition, NE is used to describe the ability of information to transmit from one node to other nodes [49]. Higher NE values means a more efficient information transfer between network nodes [50].

After 4 weeks of taVNS treatment, our study found that patients with MDD demonstrated increased DC and NE in the left AG, which was a key node of the DMN. Dysfunction of the DMN has been widely reported in patients with MDD, which is associated with the processing of self-reference, emotional appraisals, and rumination [51]. Previous studies have reported that patients with MDD had decreased nodal topological properties within the DMN [28,41,52], suggesting that the ability of information transmission was impaired in MDD. Li et al. also found that ECT can improve the depressive symptoms in patients with MDD by enhancing the NE of the DMN [22].

Wu et al. observed that after ECT intervention, patients with MDD exhibited increased DC of the DMN (precuneus and bilateral AG) [30]. Our findings were consistent with prior studies, indicating the function recovery of DMN (e.g., the AG) from the aspect of topological organization after taVNS treatment. In addition, our study found that the alternation of DC in the left AG was associated with the alternation of HAMD scores in patients with MDD after taVNS treatment. Thus, we may speculate that the taVNS may regulate the disrupted DC and NE of the DMN to achieve an antidepressant effect, which could be the potential intervention target for the treatment of patients with MDD.

### 4.4. Functional Connectivity Alterations after taVNS Treatment

In our study, after treatment with taVNS, patients with MDD exhibited reduced connectivity between the DMN–FPN, DMN–CON, and FPN–CON. The abnormality of these brain networks has been documented in previous research [53,54,55], which suggests that patients with MDD may have disruptions in multiple networks. Hence, our findings are consistent with those of the previous literature.

The DMN, a large-scale distributed network, is involved in the processing of self-reference and the regulation of affectivity [56]. The FPN, a flexible cognitive control center, is a part of the top–down control system and is essential in a wide range of cognitive processes [57]. A meta-analysis identified an imbalance in FC between the DMN and FPN in patients with MDD [58]. Previous studies have shown that ECT, antidepressant medications, and taVNS can relieve depressive symptoms by reducing FC between the DMN and FPN in patients with MDD [8,59,60]. In this study, patients with MDD had decreased FC between the DMN and FPN after 4 weeks of taVNS treatment, which is consistent with the reported findings of previous studies.

The CON, a key part of the top–down control system, is involved in the flexible control of goal-directed performance [61]. Recent neuroimaging research indicated that patients with MDD have stronger FC between the DMN and CON [62]. Wu et al. found that patients with MDD showed abnormal FC between the FPN and CON [63], and the FC was associated with the HAMD scores. Argyelan et al. indicated that after ECT intervention, patients with MDD exhibited reduced FC between the DMN and CON [64]. Moreover, Kang et al. observed that after 2 weeks of rTMS treatment, patients with MDD showed decreased FC between the FPN and CON [65]. Our findings were consistent with those of these studies, further highlighting the importance of these high-level cognitive networks (e.g., DMN, FPN, CON) in MDD. Therefore, it is our opinion that taVNS may improve the clinical symptoms of depression by downregulating the FC between DMN–FPN, DMN–CON, and FPN–CON in patients with MDD.

### 4.5. Limitations

This study has several limitations. First, the sample size was small, which may have constrained the generalizability of the results. Second, data on the number of episodes and the history of medication use were not collected. Third, taVNS is not yet widely accepted clinically; therefore, further studies are necessary to confirm the result of the present study and to verify the practicability of taVNS in clinical settings. Finally, our current study was conducted over a 4-week period, and the results may have been influenced by the relatively short washout period of 2 weeks. We intend to assess the long-term effects of taVNS therapy in a future study.

## 5. Conclusions

By combining the graph theory and NBS analysis, our study demonstrated that taVNS can modulate disrupted global (Eglob and Lp) and nodal properties (DC and NE), and that changes in network properties (Lp and DC) were correlated with changes in HAMD scores. Furthermore, patients with MDD exhibited reduced connectivity between the DMN–FPN, DMN–CON, and FPN–CON. These findings demonstrate that taVNS treatment may improve depressive symptoms by modulating the disrupted network topological organization in patients with MDD, which may provide new insights into the underlying brain mechanism for patients with MDD.

## Figures and Tables

**Table 1:** Demographic and clinical characteristics of participants.

	MDD (*n* = 19)
Sex (male/female)	13/6
Age (years)	38.89 ± 14.48
HAMD	29.11 ± 5.59
HAMA	21.00 ± 7.10
SAS	57.53 ± 9.01
SDS	66.89 ± 10.70

Abbreviations: HAMA, Hamilton Anxiety Rating Scale; HAMD, 24-Hamilton Depression Rating Scale; MDD, major depressive disorder; SAS, Self-Rating Anxiety Scale; SDS, Self-Rating Depression Scale.

**Table 2:** Changes in neuropsychological scales in patients with MDD after taVNS treatment.

	Pre	Post	*t*	*p*
HAMD	29.11 ± 5.59	15.05 ± 4.47	9.692	<0.001
HAMA	21.00 ± 7.10	10.74 ± 4.04	7.446	<0.001
SAS	57.53 ± 9.01	42.89 ± 9.67	7.664	<0.001
SDS	66.89 ± 10.70	50.84 ± 11.78	7.160	<0.001

Abbreviations: HAMA, Hamilton Anxiety Rating Scale; HAMD, 24-Hamilton Depression Rating Scale; MDD, major depressive disorder; SAS, Self-Rating Anxiety Scale; SDS, Self-Rating Depression Scale; taVNS, transcutaneous vagus nerve stimulation.

**Table 3:** Changes in topological properties in patients with MDD after taVNS treatment.

	Pre	Post	*t*	*p*
Eglob	0.269 ± 0.004	0.274 ± 0.004	−4.537	<0.001
Lp	0.790 ± 0.025	0.760 ± 0.015	6.353	<0.001
DC of left AG	18.43 ± 5.72	25.34 ± 3.71	−7.003	<0.001
Ne of left AG	0.274 ± 0.022	0.291 ± 0.025	−2.123	0.048

Abbreviations: MDD, major depressive disorder; taVNS, transcutaneous vagus nerve stimulation; Eglob, global efficiency; Lp, characteristic path length; DC, degree centrality; AG, angular gyrus; Ne, nodal efficiency.

## Data Availability

The data presented in this study are available upon request from the corresponding author due to the data not being easily accessible.

## Supplementary Materials

The following supporting information can be downloaded at: https://www.mdpi.com/article/10.3390/brainsci14090945/s1, Figure S1: Paired *t*-test of neuropsychological scales in patients with MDD before and after taVNS treatment.; Table S1: Description of topological properties.
