# Supplementary material for: Transcutaneous Auricular Vagus Nerve Stimulation Modulating the Brain Topological Architecture of Functional Network in Major Depressive Disorder: An fMRI Study
Source: Brain Sci. 2024 Sep 21;14(9):945. doi: 10.3390/brainsci14090945 (PMC11430561; doi:10.3390/brainsci14090945)
Supplement: Supplementary file 1 [file brainsci-14-00945-s001.zip › brainsci-3042450-supplementary.pdf]

Table S1 Description of topological properties

Metrics	Symbol	Description
Global network properties		
Clustering coefficient	Cp	The clustering coefficient of node i refers to the actual number of edges among all the first neighbor nodes of node i divided by the maximum possible number of edges.
Characteristic path length	Lp	The average shortest path length of node i refers to the average value of the shortest path length between node i and all other nodes. The characteristic path length (Lp) is equal to the average value of the average shortest path length of all nodes in the network, which measures the extent of the average connectivity or overall efficiency of the network.
Normalized Cp	γ	A ratio of the clustering coefficient between real and 100 random networks, which quantifies the local interconnectivity of a network.
Normalized Lp	λ	A ratio of the characteristic path length between real and 100 random networks, which quantifies the overall routing efficiency of a network.
Small-world	σ	$\Sigma = \gamma / \lambda$; Quantifies the organization of a network, with $\sigma > 1$ indicating a network has a small-world property.
Global efficiency	Eglob	The average inverse shortest path length, which measures the ability of parallel information transmission over the network.
Local efficiency	Eloc	Mean of the global efficiency of subgraphs computed on the immediate neighbors of a region, which is a measure of the fault tolerance of the network.
Nodal network properties		
Nodal efficiency	NE	NE refers to the average value of the reciprocal of the shortest pathlength between node i and all other nodes, which can measure the information dissemination ability of node i and other nodes in the network.
Degree centrality	DC	A count of the number of connections that each node has to the rest of the network.
Betweenness centrality	BC	The betweenness centrality of a node i is defined as the number of shortest paths between any two nodes that run through node i, which quantifies how much information might traverse the node.

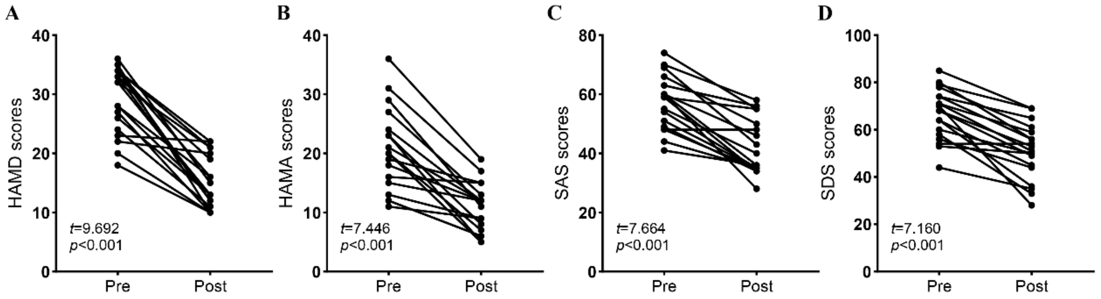

Figure S1 Paired *t-test* of neuropsychological scales in patients with MDD before and after taVNS treatment. A. HAMD scores in MDD patients before and after taVNS treatment; B. HAMA scores in MDD patients before and after taVNS treatment; C. SAS scores in MDD patients before and after taVNS treatment; D. SDS scores in MDD patients before and after taVNS treatment.

Abbreviations: MDD, major depressive disorder; taVNS, transcutaneous auricular vagus nerve stimulation; HAMD, Hamilton Depression Scale; Hamilton Anxiety Rating Scale, HAMA; Self-Rating Depression Scale, SDS; Self-Rating Anxiety Scale, SAS.
